# The Mouth in Its Relation to Stomach and Intestinal Diseases

**Published:** 1909-07-15

**Authors:** H. G. Langworthy


### THE MOUTH IN ITS RELATION TO STOMACH AND INTESTINAL DISEASES.

BY H. G. LANGWORTHY, M.D.

As expressed by Baker, the fact that "Diseased teeth are a cause of other ills of the human body has long been known to medical science, but that the control of these dental diseases is a decided advance in preventive medicine is not generally recognized." This statement is true not only of the teeth, but must be made to include the nose and throat as well. Thorough examination of the mouth, besides being essential in local oral disease, is of great value in the investigation of many general disturbances. For this reason a proper study of the mouth should comprise inspection of all structures such as the teeth, gums, lips, tongue, floor of the mouth, salivary glands, nose, and throat. Until this be done, progress, bacteriological or clinical, will be necessarily slow.

The remote effects of oral infection occur chiefly in two ways:

First, by direct absorption of toxins or bacteria into the circulation from such processes as carious teeth, inflamed gums, stomatitis, alveolar abscess, and bone necrosis; and second, by infiltration of food from putrid deposits about the gums and teeth, making a mixed infection constantly passing into other cavities, *i. e.* the throat, lungs, stomach, and intestines.

Types of the first class of cases are well illustrated in the systemic symptoms of fever, prostration, headache, and general malaise.

The second class is best seen in the relation of the mouth to gastro-intestinal and respiratory diseases to be described.

Contamination of food is important in causing decay of food within the body, the poisons of which frequently cause secondary gastric catarrh, various forms of auto-intoxication, anemia, nervous debility, and appendicitis. A foul mouth and decaying teeth, particularly in children, decidedly increase the chances of catching such contagious and infectious diseases as scarlet fever, diphtheria, measles, and tuberculosis. A clean mouth will do much to prevent tubercle bacilli gaining a foothold in the body. Wadsworth remarks: "From the hygienic standpoint the secretions of the mouth constitute the chief if not the only source of respiratory infections, and the infectious material is transferred from one person to another, in some cases through the air as from sneezing or coughing, and to an even more serious extent by personal contact or by the use in common of the various accessories of life." Certainly the connection between carious teeth and tuberculous glands in the neck as outlined in some of the clinical cases is direct. Emerson, although having no figures to prove his assertions, is sure that "mouth-infections involving the fauces and tonsils are more common in individuals with carious teeth." He suggests further that one way of checking respiratory diseases will be by the prevention and correction of dental diseases, supplemented by the use of bland alcoholic solutions as mouth-washes.

At the risk of being considered tedious, I wish to state that practically the only real germicide which is effective and yet not powerful enough to destroy soft tissues is alcohol. Most of the preparations which can be used in the mouth, as far as their germicidal qualities are concerned, owe their efficiency to the amounts of alcohol which they contain. The majority of the preparations on the market not containing alcohol are valuable as antiseptics rather than as germicides. All dentists, therefore, should use quantities of pure alcohol in disinfecting their hands and instruments.

But to return to our subject. The following case, still under observation, is interesting as demonstrating the relation between a disease of the mouth and severe gastritis:

*Case* 1. Male, forty-five years of age, carpenter. History of persistent stomach trouble for two years, gradually growing worse. Losing weight, anemic. Unable to retain food in stomach for any length of time. Gave up his work a few days ago to place himself under the care of Dr. A. H. Blocklinger of Dubuque, who called in the services of Dr. A. E. McEvoy, a dentist, and myself.

Without going into detail I will simply state that the patient had one of the worst cases of pyorrhea alveolaris which we had seen for some time. For two years, at least, pus from the mouth had been draining into the stomach, with the natural result of a severe gastric infection and general toxemia. Appropriate treatment shared in by the physician, dentist, and myself, has already begun to result in improvement.

The following case I have taken from Ewald ("Diseases of the Stomach"). Note that the doctor, a stomach specialist, does not hesitate to attribute many of these diseases to the mouth:

Male, adult; typical mucous catarrh, atificial upper plate never removed at night; cleansed about every third day. The plate was covered with a dirty-white coating consisting of numerous fungi and masses of cocci, while the hard palate was markedly reddened and dotted with small aphthous ulcers. In the slimy stomach contents there were small brown streaks which consisted of granular blood pigment and numberless fungi and yeast-cells. The patient's complaints were relatively slight, and began only after treatment by the dentist. In this case the swallowed bacteria unquestionably kept up a constant state of irritation of the gastric mucous membrane.

The above however, is not the only aspect of the subject. Leading surgeons in this country tell us that the actual danger of abdominal operations involving the intestines is increased in every case where there is the slightest chance of oral sepsis, while as eminent a medical authority as Dr. Osler writes that—"There is not any one single thing more important to the public in the whole range of hygiene than the hygiene of the mouth. If I were asked to say whether more physical deterioration was produced by alcohol or by defective teeth, I should unhesitatingly say defective teeth."

In conclusion it is necessary to add that while my remarks have been more or less restricted to the relationship of the mouth to the gastro-intestinal and respiratory tracts it must be remembered that quite a large number of mouth and throat affections may be local manifestations of a general disease—as for instance, as grouped by Stein laryngeal, tuberculosis in pulmonary tuberculosis, mucous patches in syphilis, perichondritis of the larynx in typhoid fever, coryza in measles, angina in scarlet fever, paralysis of the vocal cords in tabes, and pharyngitis sicca in diabetes mellitis.

Stein also refers to the fact that the regional disturbances create or favor the introduction of other conditions—as, for instance, a foreign body resulting in pneumonia, rhinitis sometimes resulting in erysipelas, septal ridges in asthma, adenoids resulting in epileptic equivalents. It has also been noted many times that diseased tonsils may induce rheumatism.—*Cosmos.*

